# Sleep EEG foundation models reveal within-stage microstructure that improves health screening beyond traditional stages

**DOI:** 10.21203/rs.3.rs-9044150/v2

**Published:** 2026-06-26

**Authors:** William Coon, Mattson Ogg

**Affiliations:** 1 Intelligent Systems Center, Research and Exploratory Development Department Johns Hopkins Applied Physics Laboratory; 2 Johns Hopkins University, Whiting School of Engineering

## Abstract

Sleep physiology provides rich longitudinal biosignals reflecting integrated brain and systemic physiology, yet polysomnography is commonly compressed into coarse, human-defined stages. We asked whether self-supervised foundation models learn sleep EEG structure beyond traditional staging and encode enriched health information. Using 11,261 overnight recordings, we trained transformers on unlabeled sleep data and probed representations across diagnostic, demographic and functional outcomes. Compared with architecture-matched transformers trained from random initialization on each downstream task, SSL pretraining improved performance across several outcomes. Compared with five-stage-supervised pretraining, EEG-only advantages were clearest for BMI and age, while differences for AHI, sex, and functional outcomes were smaller, nominal, or not reliable. In nested controls, EEG-derived self-supervised model scores retained incremental value beyond covariates, stage summaries, spectral summaries, and a matched five-stage representation. Embedding analyses show that models recover the stage scaffold without labels while preserving higher-resolution, stage-anchored structure that carries task-specific health information beyond the five-stage interface.

## Introduction

Sleep provides a densely informative window into human physiology, reflecting coordinated neural, cardiovascular, respiratory, metabolic, and autonomic activity across the night.^1^ To make this complex signal clinically tractable, sleep medicine has long compressed polysomnography (PSG) into a small set of visually scored stages—Wake, N1, N2, N3, and REM—based on criteria originally formalized by Rechtschaffen and Kales and later refined by the American Academy of Sleep Medicine^2–4^. This five-stage interface remains foundational to clinical sleep medicine and provides a necessary reference point for new data-driven representations.

Yet the same compression that makes conventional staging clinically useful also makes it intentionally lossy. Visual scoring is subjective and variable across raters^5,6^, fixed heuristics can obscure individual- and age-related physiology^7^, and 30-s epochs impose a coarse temporal grid that limits sensitivity to shorter-lived and within-stage dynamics^8–10^. These limitations also propagate into automated scoring systems that largely learn to reproduce human-defined labels, despite major advances in sleep-stage classification^11–19^. A parallel literature has therefore treated sleep as a continuum rather than a strictly discrete taxonomy, including early automated quantification and sleep/wake continuum analyses, microstate and feature-clustering approaches, whole-brain transition analyses, and recent aperiodic/fractal-cycle definitions of sleep structure^20,21,8,22,9,23–25^. Together, this work suggests that the question is not whether stages are meaningful, but what physiological information is collapsed by the five-stage scaffold.

This question is increasingly important for digital medicine because sleep is both routinely measurable and biologically integrative. Unlike many clinic-bound assays, sleep can be captured passively over long time scales with scalable sensors, making it a natural substrate for decentralized monitoring and biomarker discovery^26–30^. Its relevance is not limited to sleep disorders: sleep is linked to cardiovascular, cognitive, neurologic, and mental-health outcomes^1,31–33^. Thus, model-derived sleep representations may be useful not only when they predict immediately actionable clinical labels, but also when they summarize latent physiology. Sleep-EEG brain age is a clear example: chronological age is already known, but the gap between EEG-predicted age and chronological age provides a brain-age / brain-age-index biomarker associated with cognition, dementia risk, mortality, and broader brain health^34–37^. These developments motivate models that can learn health-relevant structure from sleep physiology without relying exclusively on predefined scoring rules.

Foundation models and self-supervised learning (SSL) offer one route to such representations. SSL can learn from large unlabeled physiological datasets by exploiting intrinsic signal structure rather than externally supplied labels^38–40^. In sleep, this strategy is especially attractive because physiology evolves continuously across time, space, and state, with local and regional dynamics that are not fully captured by categorical stage labels^41–43^. Recent sleep foundation models such as SleepFM and SleepGPT, and related representation-learning approaches such as SOM-CPC and others, have demonstrated that learned sleep embeddings can recover the canonical stage scaffold while preserving additional structure relevant to disease, age, apnea, and other phenotypes^25,44–47^.

However, these impressive results leave open a mechanistic and clinically important question: to what extent do foundation model embeddings improve prediction simply by capturing the same coarse information summarized by the five-stage scaffold (e.g., stage composition and stage-transition dynamics), versus by uncovering additional, finer-grained organization *within* stages that carries health-relevant signal? Resolving this distinction matters for both translation and discovery, because it determines whether improvements represent a repackaging of established sleep architecture or reflect previously unmeasured microstructure, and because it motivates explicit benchmarks and analyses that can isolate, summarize, and interpret any structure learned beyond the canonical stage interface.

In this study, we train Sleep2.0, a HuBERT-style transformer foundation model^48^, to test whether foundation models trained with SSL learn representations of sleep that extend beyond traditional staging, and we explicitly interrogate whether any predictive gains reflect five-stage architecture versus additional learned microstructure. Using publicly available polysomnographic datasets comprising 11,261 overnight recordings, we pretrain transformer-based models on unlabeled sleep physiology, focusing our primary analyses on EEG-only representations to align with scalable, home-relevant sensing. We then benchmark learned embeddings against two matched controls—an otherwise identical model pretrained to predict five-stage labels, and a model trained from scratch—to isolate whether downstream gains reflect the internalization of the canonical stage scaffold or additional structure uncovered through self-supervision. To establish clinical relevance, we evaluate participant-level screening behavior using discrimination, calibration, decision-analytic utility, and subgroup robustness, and we test whether EEG-derived scores retain incremental predictive value beyond demographic covariates, stage summaries, spectral summaries, staging-derived sleep-report summaries, and a matched five-stage representation. Finally, we interrogate what the models learn by quantifying and visualizing their latent organization, mapping data-driven microstates and cross-subject meta-states back to both the five-stage scaffold and familiar, biomarker-relevant EEG physiology. This integrative evaluation connects performance to mechanism, showing how EEG foundation models can preserve the familiar clinical organization of sleep while revealing higher-resolution, stage-anchored structure—most clearly N2-linked for apnea- and BMI-related information, with age-related signal distributed more broadly—that carries measurable information not captured by conventional stage summaries.

## Results

**Figure 1 |:**
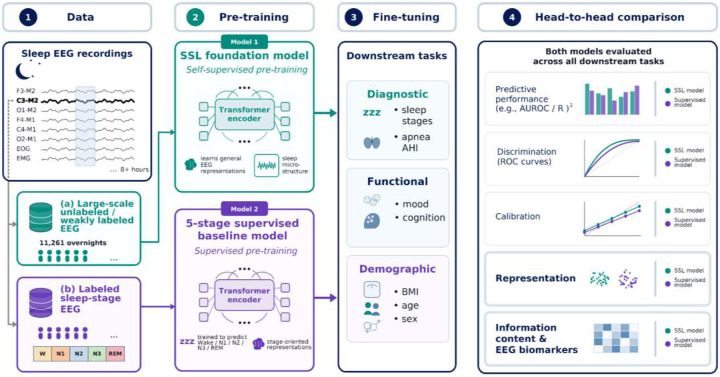
Study overview: self-supervised sleep EEG pretraining, downstream probing, and head-to-head evaluation. Sleep EEG recordings from 11,261 overnight studies were used to train and evaluate two architecture-matched transformer models. The primary Sleep2.0 model was pretrained with self-supervised learning to learn general EEG representations and sleep microstructure without sleep-stage labels. A matched supervised baseline was pretrained to predict conventional five-stage sleep labels. Both models were then fine-tuned and evaluated across diagnostic outcomes, including alertness, sleep staging, and apnea; demographic/trait-associated outcomes, including BMI, age, and sex; and functional outcomes, including mood and cognition. Downstream comparisons assessed predictive performance, discrimination, calibration, representation content, and electrophysiological/information content—with a particular focus on the latter two—to determine what SSL learns beyond the conventional five-stage interface.

To isolate whether self-supervised learning (SSL) captures health-relevant sleep structure beyond the traditional five-stage interface, we evaluated a foundation model (Sleep2.0; Figure 1) against two strictly matched control architectures: a supervised model pretrained to predict five-stage sleep labels, and a baseline model trained from scratch without pretraining.

**Figure 2 |:**
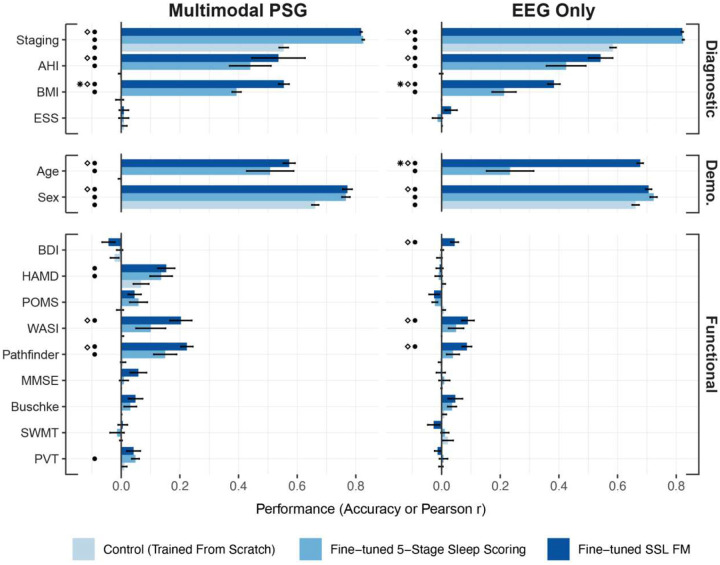
Self-supervised foundation models encode more health-relevant information from sleep EEG than supervised or non-pretrained models. Fine-tuning performance on the APPLES dataset comparing models trained from scratch, models pretrained to predict traditional five-stage sleep labels, and self-supervised (SSL) foundation models. Outcomes are grouped by intended use: diagnostic sleep metrics, demographic variables, and functional measures of mood and cognition. Dots indicate model–outcome pairs whose fine-tuning performance exceeds chance (chance = 20% for 5-class staging, 50% for binary classification, and 0 correlation for continuous outcomes), using FDR–adjusted *q*-values across the 45 SSL and baseline EEG-only models evaluated against chance (*q* < 0.05). Diamonds denote outcomes where the SSL model significantly outperforms the from-scratch baseline (paired, fold-wise Wilcoxon signed-rank test; nominal *p* with FDR correction across the 15 outcomes, *q* < 0.05). Asterisks denote outcomes where the SSL model significantly outperforms the five-stage–pretrained baseline (paired, fold-wise Wilcoxon signed-rank test; nominal *p* with FDR correction across the 15 outcomes, *q* < 0.05). Error bars show standard error of the mean across folds. For transparency, we report nominal *p* values in the text and use *q* to denote FDR–adjusted significance. Across outcomes, SSL pretraining improved performance relative to from-scratch training for several tasks. Relative to the matched five-stage-pretrained model, FDR-corrected EEG-only advantages were clearest for BMI and age, whereas AHI, sex, and functional outcomes showed smaller, nominal, or non-reliable differences. Cross-validation was performed at the participant level (all segments and all nights per participant assigned to a single fold) to prevent information leakage.

Unless otherwise noted, all analyses use strict participant-level splits and focus on EEG-only models. We emphasize EEG-only representations because they derived the greatest relative benefit from SSL pretraining and are the most broadly applicable to scalable, at-home monitoring (though multimodal PSG variants showed comparable qualitative patterns; Figure 2).^73,74,19,75,76^ To clarify clinical and translational relevance, downstream prediction targets are grouped into three intended-use categories: diagnostic sleep metrics, demographic variables, and functional outcomes.

This design separates architecture from training objective. The SSL and five-stage-supervised models share the same transformer backbone and training corpus, but differ in pretraining targets: masked pseudo-label prediction from unlabeled physiology versus supervised five-stage label prediction. Downstream tasks are therefore compared after identical task-head replacement and fine-tuning, allowing differences to be attributed to learned representation content rather than model class alone.

### Health Information is Extensively Encoded in SSL-Derived Sleep Representations

Fine-tuning the EEG-only Sleep2.0 SSL foundation model yielded above-chance prediction across a diverse set of diagnostic, demographic, and functional outcomes (Figure 2, EEG-only). Across the 15 EEG-only outcomes shown in Figure 2, we controlled the false discovery rate using Benjamini–Hochberg correction and report nominal p-values alongside FDR-adjusted q-values. After FDR correction across outcomes, SSL-derived predictions were reliably above chance for key diagnostic and demographic targets, including sleep-stage classification (82.1% accuracy; p = 9.77×10^−4^, q = 2.44×10^−3^), apnea severity (AHI, Pearson r = 0.542; p = 9.77×10^−4^, q = 2.44×10^−3^), age (r = 0.677; p = 9.77×10^−4^, q = 2.44×10^−3^), sex (70.6% accuracy; p = 9.77×10^−4^, q = 2.44×10^−3^), and BMI (r = 0.383; p = 9.77×10^−4^, q = 2.44×10^−3^). Effects for mood and cognition were smaller but still detectable for selected outcomes, including BDI (r = 0.045; p = 4.58×10^−3^, q = 8.58×10^−3^), WASI-IQ (r = 0.090; p = 2.93×10^−3^, q = 6.28×10^−3^), and Pathfinder (r = 0.086; p = 9.77×10^−4^, q = 2.44×10^−3^). Other functional measures did not exceed chance after FDR correction (q > 0.05), indicating that functional information is present but weaker and outcome-dependent in nocturnal sleep EEG.

The relatively weak performance of from-scratch models for some participant-level outcomes is expected because the same high-capacity architecture is trained only on the smaller APPLES fine-tuning cohort, whereas pretrained models first learn sleep structure from the full 11,261-recording corpus. Thus, SSL pretraining supplies both a better initialization and a more stable inductive bias for outcomes whose EEG correlates are distributed across the night. Consistent with this interpretation, smaller 2-layer and 6-layer architecture controls showed qualitatively similar pretrained-model patterns and relatively stronger from-scratch behavior when model capacity was better matched to available labeled data (Supplementary Figure 1).

To determine whether gains reflect SSL-derived representations rather than the transformer architecture alone, we compared matched models trained from scratch versus SSL-pretrained using paired Wilcoxon signed-rank tests across folds (Figure 2; nominal p-values with FDR correction across outcomes). SSL pretraining significantly improved performance over from-scratch training for sleep staging (V = 55, p = 1.95×10^−3^, q = 5.86×10^−3^), AHI (V = 55, p = 1.95×10^−3^, q = 5.86×10^−3^), BMI (V = 55, p = 1.95×10^−3^, q = 5.86×10^−3^), age (V = 55, p = 1.95×10^−3^, q = 5.86×10^−3^), sex (V = 51, p = 1.37×10^−2^, q = 2.56×10^−2^), BDI (V = 45, p = 9.15×10^−3^, q = 1.96×10^−2^), WASI-IQ (V = 53, p = 5.86×10^−3^, q = 1.46×10^−2^), and Pathfinder (V = 55, p = 1.95×10^−3^, q = 5.86×10^−3^). The same pattern of results was observed for different size configurations of the from-scratch model (Supplementary Figure 1A).

We next tested whether SSL’s advantage depends on learning sleep structure beyond the conventional five-stage scaffold by comparing against a supervised model pretrained for five-stage sleep scoring (paired Wilcoxon signed-rank tests; FDR correction across outcomes). After FDR correction, SSL significantly outperformed five-stage pretraining for BMI (V = 55, p = 1.95×10^−3^, q = 2.93×10^−2^) and age (V = 54, p = 3.91×10^−3^, q = 2.93×10^−2^). For BDI (V = 48, p = 3.71×10^−2^, q = 0.139) and Pathfinder (V = 47, p = 4.88×10^−2^, q = 0.146), differences were nominal (p < 0.05) but did not survive FDR correction. No reliable differences were observed for AHI (V = 44, p = 0.105, q = 0.176), sex (V = 11, p = 0.105, q = 0.176), or WASI-IQ (V = 41, p = 0.193, q = 0.290). As expected, five-stage pretraining yielded slightly higher staging accuracy than SSL (82.4% vs 82.1%; V = 6, p = 2.73×10^−2^, q = 0.137), although this difference did not survive FDR correction. This pattern was consistent across pretrained models of different sizes (Supplementary Figure 1B). Together, these comparisons indicate that conventional staging captures meaningful coarse-grained structure (supporting competitive performance for several outcomes), while SSL representations encode additional health-relevant information—most consistently for age and BMI in EEG-only analyses—that is not preserved by the five-stage interface. Although chronological age is directly observable and its inference from EEG may appear trivial at first glance, accurate age prediction from sleep EEG serves as a probe of whether the representation captures age-sensitive neurophysiology. This is particularly relevant because the residual between EEG-estimated age and chronological age forms the basis of brain-age-gap biomarkers, which have recently been associated with dementia risk in longitudinal cohorts.

### Representations support participant-level screening behavior

**Figure 3 |:**
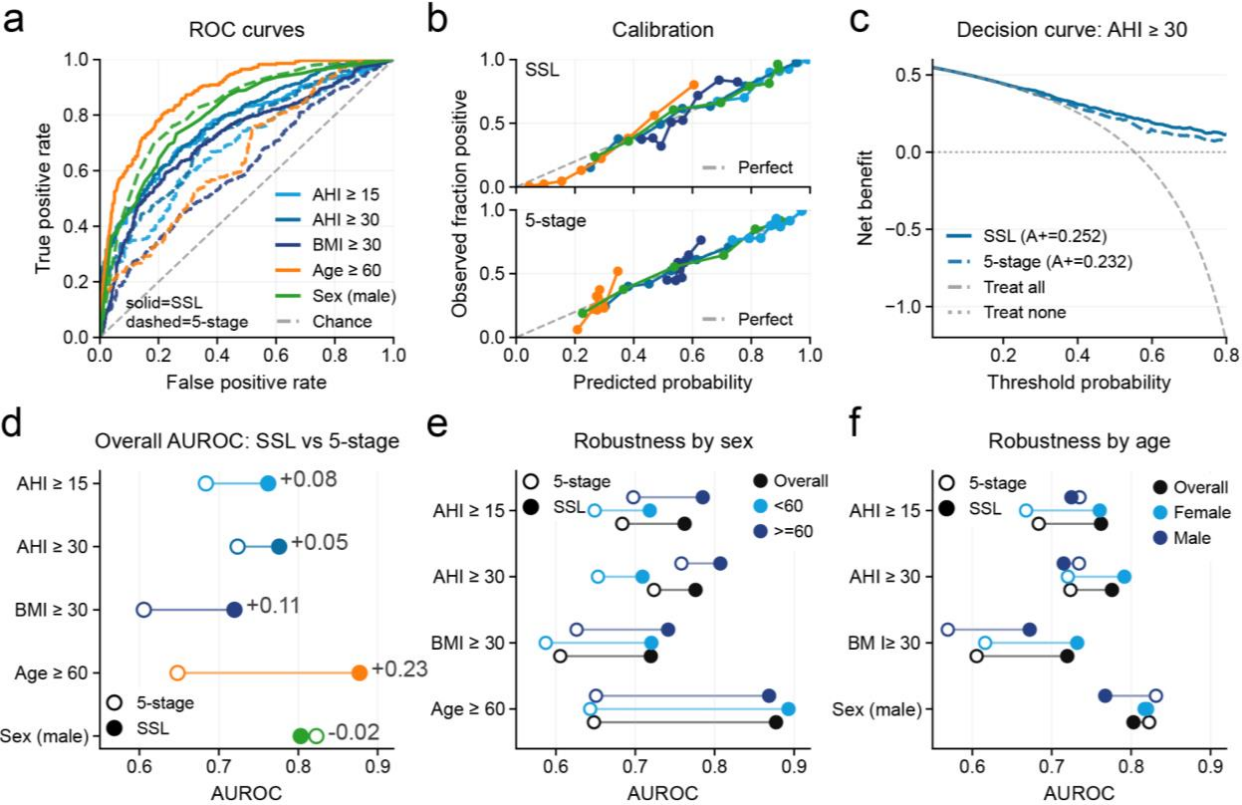
Subject-level screening evaluation from EEG-only foundation model outputs. Segment-level predictions were aggregated to the participant level (mean across 50.5-min segments per subject) and evaluated on all (n=1,044) held-out APPLES participants across representative diagnostic and demographic outcomes. (a) Receiver operating characteristic (ROC) curves for apnea severity (AHI ≥ 15; AHI ≥ 30), BMI ≥ 30, Age ≥ 60, and Sex (male). (b) Calibration curves after out-of-fold probability mapping. (c) Decision curve analysis (DCA) for AHI ≥ 30 showing net benefit across threshold probabilities relative to treat-all and treat-none strategies. (d) AUROC summary across outcomes. (E–F) Robustness analyses reporting AUROC stratified by sex (e) and by age bin (<60 vs ≥60) (f) for the indicated outcomes, shown alongside overall cohort performance.

To evaluate performance in a screening-like setting, we aggregated segment-level model outputs to the participant level (mean across segments per participant) and assessed discrimination, calibration, decision-analytic utility, and subgroup robustness (Figure 3). Across held-out participants (APPLES dataset; n = 1,044), EEG-derived scores showed moderate-to-strong discrimination for apnea severity (AUROC = 0.762 for AHI ≥ 15; AUROC = 0.776 for AHI ≥ 30), obesity (AUROC = 0.720 for BMI ≥ 30), and demographic outcomes (AUROC = 0.877 for age ≥ 60; AUROC = 0.803 for sex).

After out-of-fold probability mapping, calibration was stable across outcomes (Brier scores: 0.107 for AHI ≥ 15, 0.191 for AHI ≥ 30, 0.218 for BMI ≥ 30, 0.133 for age ≥ 60, 0.167 for sex). Decision curve analysis for severe apnea (AHI ≥ 30) showed positive net benefit over treat-all and treat-none strategies across clinically plausible threshold probabilities. Stratified AUROCs by sex and by age bin (<60 vs ≥60) demonstrated broadly similar performance across subgroups (Figure 3e,f).

### SSL-derived scores add information beyond demographics and stage-derived sleep reports

**Figure 4 |:**
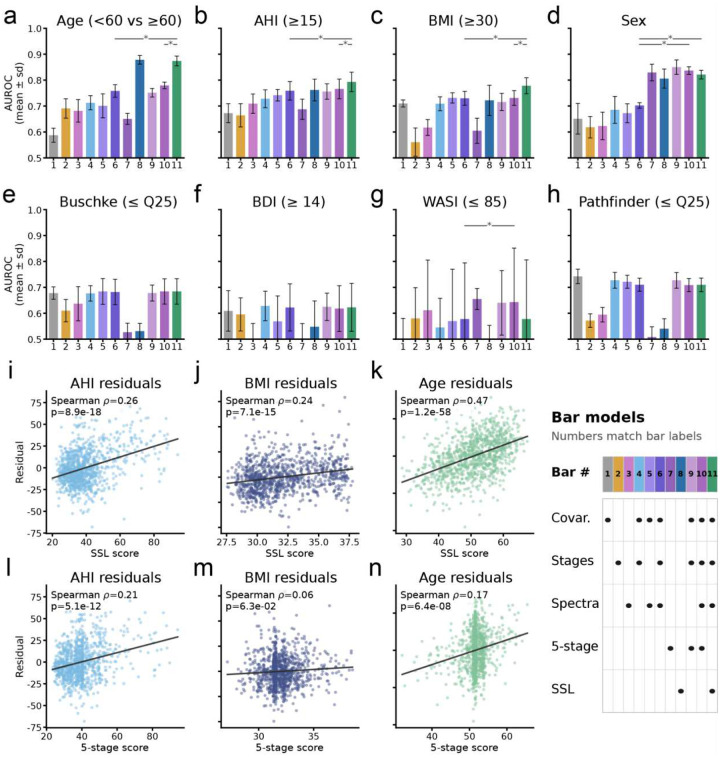
SSL-derived EEG scores retain incremental information beyond demographic covariates, conventional stage summaries, spectral summaries, and a supervised five-stage representational baseline. Participant-level screening models were evaluated in APPLES using out-of-fold 5-fold cross-validation. Bars show mean AUROC – s.d. across folds for eight thresholded outcomes: **(a)** age ≥60 years, **(b)** AHI ≥15, **(c)** BMI ≥30, **(d)** sex, **(e)** Buschke Selective Reminding Test ≤ lower quartile, **(f)** BDI ≥14, **(g)** WASI-IQ ≤85, and **(h)** Pathfinder ≤ lower quartile. The numbered bars correspond to the feature blocks shown in the inset: **1**, covariates only; **2**, conventional sleep-stage summary features only; **3**, conventional spectral summary features only; **4**, covariates + stage summaries; **5**, covariates + spectral summaries; **6**, covariates + stage summaries + spectral summaries; **7**, target-specific score from the matched five-stage-pretrained model; **8**, target-specific SSL score; **9**, covariates + stage summaries + five-stage score; **10**, covariates + stage summaries + spectral summaries + five-stage score; and **11**, covariates + stage summaries + spectral summaries + SSL score. Covariates were outcome-specific and excluded the target variable to avoid circularity. Stage summaries were derived from ground-truth AASM staging and included sleep-efficiency, latency, stage-composition, transition, and bout-duration features. Spectral summaries included whole-night and NREM band-power, relative-power, slowing-ratio, frequency-summary, and aperiodic 1/f-slope features. Models containing both SSL and five-stage scores were not fit, so bars 10 and 11 compare the two representational baselines against the same non-transformer feature set. Brackets indicate planned paired fold-wise comparisons using exact sign-flip tests; asterisks denote p < 0.05; selected ROC curves with bootstrap confidence intervals shown in Supplementary Figure 2. The rightmost two fully adjusted learned-representation models are bar 10 (covariates + stages + spectra + five-stage score) and bar 11 (covariates + stages + spectra + SSL score). **(i–k)** Partial-residual associations for continuous AHI, BMI, and age after regressing out covariates, stage summaries, and spectral summaries using out-of-fold ridge regression. Residual outcome variance remained associated with the SSL score for AHI, BMI, and age, indicating information not captured by conventional covariates, stage summaries, or spectral summaries alone. **(l–n)** Same as i-k, but for the 5-stage-pretrained representations.

Because several targets are correlated with demographic risk factors, sleep architecture, and conventional spectral physiology, we next tested whether EEG-derived SSL scores retained information beyond simpler explanatory features. We compared participant-level screening models using covariates, ground-truth stage summaries, conventional EEG spectral summaries, the target-specific score from the matched five-stage-pretrained model, the target-specific SSL score, and prespecified combinations of these blocks (Figure 4a–h). No model included both SSL and five-stage scores, allowing each learned representation to be compared against the same non-transformer baseline.

Spectral summaries were informative, especially for age (spectra-only AUROC = 0.680), and also carried signal for AHI (0.677) and BMI (0.608). Adding spectra to covariates + stage summaries improved AUROC for age (0.738 to 0.781), AHI (0.710 to 0.717), and BMI (0.710 to 0.728), confirming that classical spectral structure explains part of the signal. However, the fully adjusted SSL model still exceeded the fully adjusted five-stage representation for age (0.880 vs 0.799), BMI (0.776 vs 0.730), and AHI (0.761 vs 0.733), whereas sex slightly favored the five-stage representation (0.834 vs 0.846).

In matched residual analyses after regressing out covariates, stage summaries, and spectral summaries, SSL scores remained associated with residual age (Spearman ρ = 0.47, p = 1.2×10^−58^), AHI (ρ = 0.26, p = 8.9×10^−18^), and BMI (ρ = 0.24, p = 7.1×10^−15^). The corresponding five-stage scores showed weaker associations for age (ρ = 0.17, p = 6.4×10^−8^), AHI (ρ = 0.21, p = 5.1×10^−12^), and BMI (ρ = 0.06, p = 6.3×10^−2^). Thus, SSL retained incremental information beyond covariates, stages, spectra, and the matched five-stage representational baseline, most clearly for age and BMI and more modestly for AHI.

### PSG context analysis

As an additional context analysis, a multimodal PSG model (EEG, EOG, EMG, ECG, respiration) modestly improved discrimination for severe apnea and BMI, consistent with added respiratory and peripheral physiology, while EEG-only remained competitive and retained stronger performance for age and mild apnea thresholds (Supplementary Figure 3; Supplementary Table 2).

### SSL learns the gross geometry of conventional stages without stage labels

**Figure 5 |:**
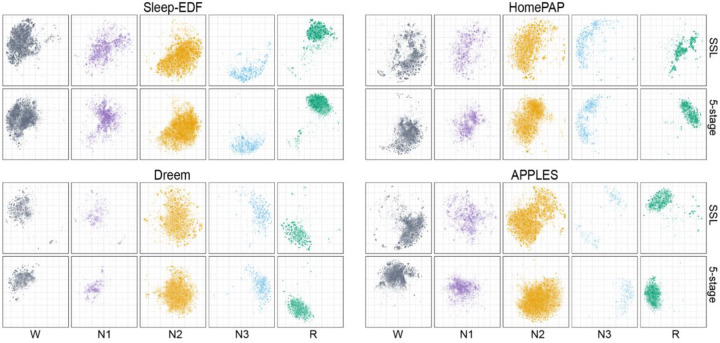
SSL-trained models recover the canonical sleep-stage scaffold across datasets. Two-dimensional t-SNE visualizations of final-layer EEG embeddings from Sleep-EDF, HomePAP, Dreem, and APPLES (displayed are up to n=151 records sampled at random from each, for visual clarity). For each dataset, embeddings are shown for the SSL-pretrained model and the matched five-stage-supervised baseline, faceted by ground-truth AASM stage: Wake, N1, N2, N3, and REM. Although the SSL model was pretrained without sleep-stage labels, its embeddings organize epochs into stage-specific regions that broadly mirror the supervised five-stage representation across all four datasets. At the same time, SSL embeddings retain visible within-stage dispersion, consistent with the idea that SSL recovers the canonical stage scaffold while preserving additional structure collapsed by five-stage supervision.

Given that five-stage pretraining remained competitive for some outcomes, we next asked whether the SSL embedding space recovers canonical stage organization despite using no stage labels during pretraining. Two-dimensional t-Distributed Stochastic Neighbor Embedding (t-SNE^77^) projections of epoch embeddings prior to any fine-tuning, colored by ground-truth stage labels, showed that SSL embeddings reproduce the expected global stage geometry in all four held-out test datasets (Figure 5). This cross-dataset consistency underscores the generalizability of the SSL model’s latent representation and demonstrates that it autonomously captures the primary physiological distinctions underlying traditional sleep stages while also supporting richer internal structure.

We used each held-out dataset according to the labels and outcomes it supports. APPLES was the primary health-outcome cohort because it is the largest held-out dataset with rich diagnostic, demographic, mood, and cognitive measures. HomePAP is smaller but shares AHI, age, BMI, and sex, enabling external replication for those outcomes. Sleep-EDF and Dreem provide stage-labeled EEG benchmarks but lack comparable participant-level health metadata. Accordingly, we used all four datasets for stage-geometry visualization and latent-granularity analyses, HomePAP for shared outcome replication, and APPLES for the full health-outcome analysis.

### SSL supports a higher-resolution decomposition of within-night sleep structure

We quantified the complexity of representational microstructure using a within-night Bayesian Information Criterion (BIC) cluster-sweep analysis on four held out data sets. For each recording, we applied k-means clustering to the final-layer transformer embeddings (prior to any fine-tuning) across a range of candidate state counts and identified the state number minimizing BIC (Figure 5). Across datasets, SSL supported higher selected k than the five-stage baseline: APPLES median = 11 (SSL) vs 5 (5-stage), HomePAP 10 vs 5, Dreem overall 10 vs 5, and Sleep-EDF 12 vs 5; paired tests were significant for each dataset (APPLES, HomePAP, Sleep-EDF: p < 2.2×10^−16^; Dreem: p = 4.7×10^−15^; Supplementary Figure 4). These results were stable across random initializations of the clustering procedure. Note that BIC is used here only as an operational, penalized model-selection criterion for within-record compression. Hence the BIC result is comparative rather than ontological: under the same clustering procedure, SSL embeddings support finer stage-anchored partitions than five-stage-pretrained embeddings. The usefulness of this extra granularity is evaluated separately through downstream analyses: Rosetta summaries, meta-state prediction, spectral/event-level physiology, and robustness controls.

Because microstates are estimated independently within each recording, we summarized where additional complexity concentrates by anchoring microstates to AASM stages (“Rosetta” mapping). For each subject, each microstate was assigned to the stage in which it most frequently occurred. Across recordings, Wake and N2 consistently subdivided into more microstates than N3 or REM (Supplementary Figure 4), indicating that SSL-derived resolution is not uniformly distributed across sleep but instead preferentially refines stages known to encompass heterogeneous physiology^43,78,79^. Importantly, this indicates that the increased representational complexity observed does not imply greater interpretive opacity. Rather, it reflects a higher-resolution decomposition of sleep physiology that preserves traditional stages while subdividing them into reproducible, data-driven substates.

We next tested whether this microstate/Rosetta pattern was recoverable under different k-means initializations. Holding each APPLES recording at its selected k and varying only the clustering seed, fixed-k SSL microstate assignments were recoverable across 10 seeds (mean pairwise ARI = 0.793; mean pairwise AMI = 0.856; n = 1,044 recordings). The selected-k distribution was centered near 11 clusters per recording (median = 11; mean = 11.46), and stage-anchored Rosetta counts were stable: in all 10 seeds, median microstate counts were N2 = 5, Wake = 3, REM = 1, N1 = 1, and N3 = 0. The expected Rosetta ordering held in every seed (N2 > N3, N2 > REM, Wake > N3, Wake > REM; one-sided Wilcoxon tests, all p < 5 × 10^−130^). Thus, the conclusion that SSL preferentially subdivides heterogeneous N2 and Wake structure is not dependent on a single clustering initialization.

### Embedding atlases make microstructure and phenotype gradients visually interpretable

**Figure 6 |:**
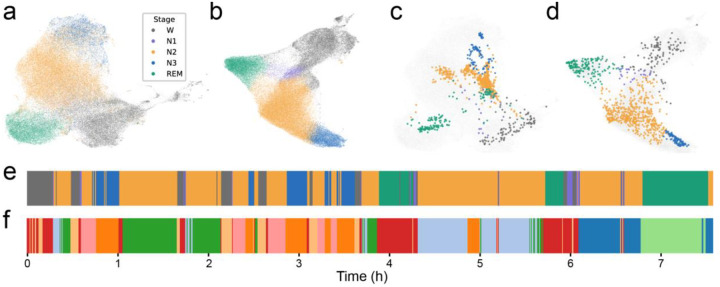
Global sleep embedding atlases and an example subject’s projections onto SSL and five-stage manifolds. **(a,b)** Two-dimensional UMAP projections (“global manifolds”) of 30-s EEG epoch embeddings from a foundation model pretrained with self-supervised learning (SSL; a) and a matched model trained with five-class sleep stage supervision (five-stage; b). Points show all epochs used to form the global atlases and are colored by five-stage sleep labels (Wake, N1, N2, N3, REM). **(c)** A representative subject’s SSL embeddings projected onto the SSL global manifold and colored by that subject’s five-stage labels, illustrating where this night’s epochs fall within the population-level SSL geometry. **(d)** The same subject’s five-stage embeddings projected onto the five-stage global manifold and colored by five-stage labels. The SSL embedding in (c) forms compact, well-separated microstate islands, whereas the stage-supervised embedding in (d) shows greater dispersion and overlap among microstates, consistent with reduced expression of fine-grained structure under five-stage supervision. **(e)** The subject’s five-stage hypnogram across the night (30-s epochs), using the same stage color scheme as panels (a–d). **(f)** The subject’s corresponding microstate timeline, using arbitrary colors; each color corresponds to one of this subject’s microstates.

To visualize how microstates organize in a shared coordinate system, we constructed global UMAP atlases from pooled APPLES epoch embeddings and, additionally, projected an exemplar subject onto each atlas (Figure 6). Stage-colored views confirmed that both representations preserve coarse stage organization, but SSL expresses clearer within-stage subdivision. When viewed within an individual subject, SSL embeddings form compact, well-separated “islands,” whereas stage-supervised embeddings of those same epochs showed greater dispersion and overlap (Figure 6c,d). Consistent with N1’s transient, low-prevalence role in typical sleep architecture, N1 epochs often do not form stable, dedicated microstates. Timeline views show that microstate progression over the course of the night exhibits structure patterning consistent with preserving gross 5-stage structure while exhibiting higher-resolution patterns within stages. Together, these atlases provide an intuitive visualization of how SSL preserves the gross sleep-stage scaffold while expressing a finer microstate geometry.

**Figure 7 |:**
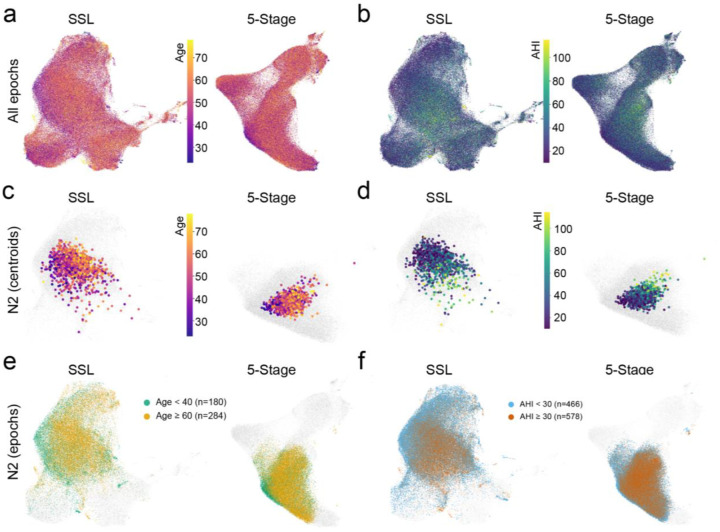
Example phenotype overlays on the global embedding atlases. **(a)** Left: SSL global manifold; right: five-stage global manifold. Colored overlays illustrate continuous phenotype structure for age. **(b)** Same as in (a), but overlaid colors represent AHI in the same cohort (APPLES). **(c)** SSL and 5-stage manifolds for N2 only shown in light gray background. Colored overlays illustrate subject-level embedding centroids from N2 sleep, colored by ground truth Age. **(d)** same as (c), but colors show subjects’ AHI scores (ground truth). **(e)** SSL and 5-stage global manifolds shown in light gray background. Colored overlays illustrate binarized phenotype (Age) from N2 sleep epoch embeddings. **(f)** Same as (e), but colors show binarized AHI in the same cohort.

To assess whether health information is expressed in manifold geometry, we overlaid phenotypes on the global atlases. Across diagnostic and demographic phenotypes (apnea severity, age, BMI, sex), both SSL and five-stage manifolds exhibited structured gradients, with SSL atlases showing additional continuous variation for several outcomes (Figure 7). When visualizations were restricted to N2 epochs, gradients became more pronounced, consistent with the Rosetta analysis indicating that much of the added resolution concentrates within N2.

To test the stability of individuals’ representations, we used Sleep-EDF, which includes 74 participants with two nights each, for an exploratory repeated-night analysis. A correlation-based night-level embedding “fingerprinting” analysis correctly matched the alternate night from the same participant in 60/148 SSL queries (40.5%; 95% CI 32.6–48.9%), above a conservative 1/74 chance baseline (exact binomial p < 2.2×10^−16^). SSL also exceeded the five-stage baseline in top-1 matching accuracy (40.5% vs 22.9%) and same-participant night-pair similarity (Wilcoxon V = 1311.5, p = 8.0×10^−4^). These results suggest a stable trait-like component while leaving substantial night-specific variation (Supplementary Figure 5).

### A cross-subject meta-state model quantifies within-stage signal beyond the five-stage scaffold

**Figure 8 |:**
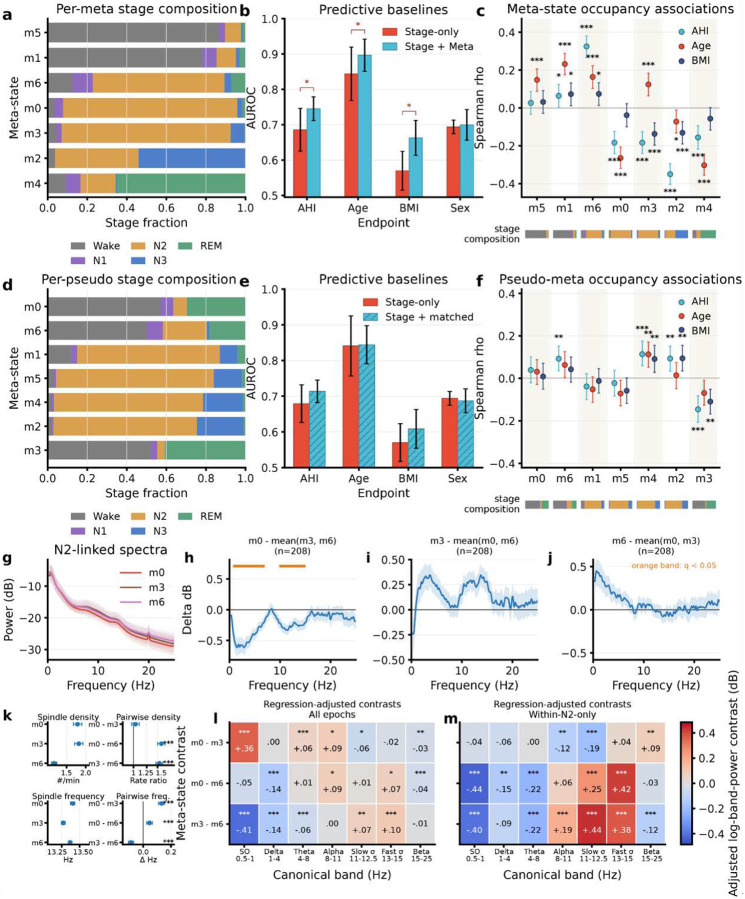
Meta-states reveal phenotype-linked, spectrally distinct N2 microstructure beyond conventional stage summaries. **(a)**, Stage composition of the fixed EEG-only M=7 APPLES meta-state solution. Stacked bars show P(stage|meta-state) for Wake, N1, N2, N3, and REM, with meta-states ordered as m5, m1, m6, m0, m3, m2, and m4. **(b)**, Cross-validated predictive baselines for binary diagnostic/demographic contrasts using five-stage sleep-architecture summaries alone (“Stage-only”) or the same summaries augmented with meta-state occupancy/diversity features (“Stage + Meta”). **(c)**, Subject-level associations between meta-state occupancy and continuous AHI, age, and BMI. Points show Spearman ρ, vertical intervals show 95% confidence intervals, and the stage-composition strip below the axis repeats the stage profile of each meta-state. Asterisks denote Benjamini–Hochberg FDR-corrected significance across meta-states within each phenotype (*q<0.05, **q<0.01, ***q<0.001). **(d–f)**, Forced M = 7 control analysis using embeddings from the matched five-stage-pretrained model. The same meta-state pipeline was applied to the five-stage representation to preserve the clustering procedure, nominal state count, and feature dimensionality. **(d)**, Stage composition of the resulting five-stage pseudo-meta-state solution. **(e)**, Cross-validated binary prediction using five-stage summaries alone versus summaries augmented with five-stage pseudo-meta-state occupancy/diversity features, plotted as in panel b. **(f)**, Subject-level associations between five-stage pseudo-meta-state occupancy and continuous AHI, age, and BMI, plotted as in panel c. This control did not reproduce the SSL stage+meta gains or the same organized phenotype-association structure, indicating that the SSL results are not explained simply by adding features or applying the same clustering pipeline to any transformer embedding. **(g)**, Mean spectra for the three N2-linked meta-states selected from the stage-composition analysis (m0, m3, and m6); shading denotes across-subject variability. **(h–j)**, Pairwise within-subject spectral contrasts between N2-linked meta-states, plotted as Δ power in dB for m0–m3, m0–m6, and m3–m6. Numbers in panel titles indicate the number of subjects contributing valid spectra for both meta-states in the contrast. Orange bars indicate frequency bins surviving FDR correction across frequency bins (q<0.05). (k), Event-level NREM physiology for the three N2-linked meta-states, restricted to epochs scored as AASM N2. Spindle density differed strongly across meta-states (Poisson GEE omnibus χ^2^ = 294.61, p = 1.06×10^−64^, q = 9.04×10^−64^), with m6 showing lower spindle density than m0/m3. Slow-oscillation density also differed across meta-states (χ^2^ = 16.20, p = 3.04×10^−4^, q = 3.69×10^−4^). **(l,m)**, Regression-adjusted canonical-band contrasts among N2-linked meta-states using all contributing epochs (l) or only epochs scored as AASM N2 (m). Heatmap values show adjusted log-band-power contrasts in dB; positive values indicate greater adjusted band power in the first meta-state named in the row. Models included subject fixed effects and aperiodic spectral covariates. Canonical bands were slow oscillation (SO, 0.5–1 Hz), delta/slow-wave activity (1–4 Hz), theta (4–8 Hz), alpha (8–11 Hz), slow sigma (11–12.5 Hz), fast sigma (13–15 Hz), and beta (15–25 Hz). Stars indicate BH-FDR-corrected significance across the canonical-band contrast family. Persistence of spectral differences in the within-N2-only analysis supports the interpretation that N2-linked meta-states capture electrophysiologically distinct within-stage structure, rather than only mixtures of neighboring AASM stages.

Because microstate labels are recording-specific, we next mapped subject-level microstructure to a shared vocabulary by clustering microstate representatives into meta-states and summarizing each subject by meta-state occupancy/diversity. The first step estimates recording-specific within-night structure (including per-recording state granularity) and compresses it into compact representations suitable for cross-subject mapping, whereas the second step aligns those recording-specific substates into a shared cross-subject vocabulary. We chose this two-step design because it preserves local within-night structure, avoids forcing a single global K on all recordings, reduces domination of the clustering objective by the most prevalent epochs/stages, and yields subject-level meta-state occupancy features that are directly comparable across individuals. A stability-constrained M sweep supported M = 7 as a compact, interpretable solution on a broader performance plateau (Supplementary Figure 6), and the resulting meta-states were stage-anchored mixtures (Figure 8a). We treat M (the objectively determined number of common meta-states) as a resolution parameter rather than a fixed biological state count.

At M = 7, adding meta-state occupancy features to five-stage summary features improved both screening and continuous-outcome prediction for key diagnostic and demographic outcomes. For continuous outcomes, mean R^2^ increased from 0.139 to 0.211 for AHI (ΔR^2^ +0.072) and from 0.140 to 0.210 for age (ΔR^2^ +0.070) (Supplementary Figure 7). In contrast, meta-state summaries provided limited or inconsistent benefit for mood/cognition outcomes at this level of aggregation. For screening, mean AUROC increased from 0.686 to 0.745 for AHI (ΔAUROC +0.059), from 0.570 to 0.663 for BMI (ΔAUROC +0.093), and from 0.844 to 0.897 for age (ΔAUROC +0.053), with minimal change for sex (ΔAUROC +0.006) (Figure 8b).

Sensitivity analyses showed that this interpretation was stable at the stage-anchored and downstream levels, although not at the level of exact unsupervised labels. Across 10 M = 7 meta-state seeds, exact label recoverability was moderate (mean pairwise ARI = 0.513 – 0.086; range = 0.359–0.691), but matched stage-composition structure was highly stable (dominant-stage agreement = 0.879 – 0.086; stage-composition cosine similarity = 0.964 – 0.024) (Supplementary Figure 8). Repeating downstream stage+meta models across seeds yielded positive gains over stage-only summaries in 10/10 seeds for AHI, AHI ≥ 15, Age, Age ≥ 60, and BMI ≥ 30, and in 9/10 seeds for continuous BMI; sex remained small and inconsistent. Local sensitivity analyses over M = 5–9 and PCA dimensionality = 10, 20, and 50 similarly retained positive gains in all 45 seed/parameter configurations for AHI, AHI ≥ 15, Age, Age ≥ 60, BMI, and BMI ≥ 30, but not for sex (29/45 positive) (Supplementary Figure 9). Thus, M = 7 is best interpreted as a representative solution on a stability-performance plateau rather than a unique state count.

Because five-stage features already capture overall stage composition and coarse transition dynamics, any additional gain from meta-states could arise either from residual differences in global architecture (e.g., more time awake) or from higher-resolution heterogeneity within stages that the canonical taxonomy collapses. To further isolate the source of the incremental signal, we compared stage-only models against augmented models that replace global meta-state occupancy with stage-conditional occupancy features computed within N2 and within Wake. Conceptually, these conditional features describe how a subject’s N2 (or Wake) time is distributed across meta-states, independent of the total amount of N2 (or Wake) present.

Across key outcomes, conditioning meta-state occupancy on N2 preserved much of the full stage+meta gain, whereas conditioning on Wake produced smaller and less consistent gains (Supplementary Figure 10). Dominant-stage ablations provided convergent but less stable localization: excluding N2-dominant meta-state features attenuated gains in the representative M = 7 solution, but seed and local-hyperparameter checks showed that N2-vs-Wake ablation margins were small and configuration-dependent (Supplementary Figure 10). We therefore interpret these analyses as evidence that within-N2 heterogeneity contributes substantially to the SSL advantage, not as proof that a fixed N2-dominant subset uniquely explains all incremental information.

We next tested whether apparent N2 localization simply reflected N2 occupying the largest fraction of the night. Stage-conditioned controls recomputed meta-state occupancy within each canonical stage and compared the resulting gain with matched-size random feature blocks evaluated under the same folds and seeds. N2-conditioned features recovered substantial gain for apnea- and BMI-related outcomes beyond both total N2 duration and matched random feature additions: for continuous outcomes (Supplementary Figure 11), N2 was the top-performing stage-conditioned block in 9/10 seeds for AHI and 8/10 seeds for BMI, whereas age was more strongly distributed outside N2, with N1-linked features top-performing in 10/10 seeds. For binary screening (Supplementary Figure 12), N2 was top-performing in 8/10 seeds for AHI ≥ 15 and 5/10 seeds for BMI ≥ 30, while age ≥ 60 and sex showed weaker or more distributed stage specificity. Thus, within-N2 heterogeneity is an important contributor, especially for apnea- and BMI-related information, but the SSL advantage is not exclusively N2-driven across all outcomes.

To further interpret what aspects of within-stage structure relate to phenotype, we examined univariate associations between each meta-state’s subject-level occupancy and each continuous outcome (Spearman ρ; FDR correction across meta-states within each phenotype; Figure 8c). At M = 7, meta-states were stage-anchored mixtures, and the strongest phenotype associations were concentrated in N2- and Wake-dominant meta-states. Importantly, the three N2-dominant meta-states (m0, m3, m6) exhibited heterogeneous—and sometimes opposing—relationships with both apnea severity and age, implying that collapsing heterogeneous N2 physiology into a single stage can attenuate or erase phenotype signal at the five-stage level. For apnea severity, an N2-dominant meta-state (m6) was positively associated with AHI (ρ = +0.325, p = 4.54×10^−27^), while two other N2-dominant meta-states were negatively associated (m0: ρ = −0.184, p = 2.25×10^−9^; m3: ρ = −0.184, p = 2.20×10^−9^; Figure 8c); a deep-sleep–dominant meta-state also decreased strongly with AHI (m2: ρ = −0.350, p = 1.91×10^−31^). For age, Wake-dominant meta-states increased (m1: ρ = +0.232, p = 3.14×10^−14^; m5: ρ = +0.148, p = 1.46×10^−6^) while the REMdominant meta-state decreased (m4: ρ = −0.303, p = 1.34×10^−23^), and within N2 we again observed opposing directions (m6: ρ = +0.164, p = 1.07×10^−7^; m3: ρ = +0.124, p = 5.84×10^−5^; m0: ρ = −0.265, p = 3.36×10^−18^; Figure 8c). For BMI, we likewise observed both positive and negative associations across N2-linked meta-states (m6; ρ = +0.074, p = 0.016 versus m2; ρ = −0.131, p = 1.70×10^−5^; m3; ρ = −0.137, p = 6.74×10^−6^). These univariate associations were consistent with the predictive controls. Adding SSL meta-state summaries to five-stage summaries improved screening for AHI (AUROC 0.686→0.745), BMI (0.570→0.663), and age (0.844→0.897), with little change for sex (ΔAUROC = +0.006; Figure 8b). A forced M = 7 analysis applied to the matched five-stage-pretrained representation did not reproduce the same gains or organized phenotype associations (Figure 8d–f), indicating that the result is not explained simply by adding features or applying the same clustering pipeline to any transformer embedding (see also Supplementary Figure 13).

We next asked whether these phenotype-associated N2 subdivisions corresponded to recognizable EEG physiology. The three N2-linked meta-states differed in low-frequency and sigma structure (Figure 8g–m). Within AASM-scored N2 in these meta-states, m6 showed greater power in the .5–1.25Hz band (+0.40–0.44 dB) and theta power (+0.22 dB) than m0/m3, but lower slow-sigma (−0.25–0.44 dB) and fast-sigma power (−0.38–0.42 dB), arguing against simple stage admixture. Event analyses confirmed this physiological separation: spindle density differed strongly across N2-linked meta-states (χ^2^ = 294.61, p = 1.06×10^−64^, q = 9.04×10^−64^), with m6 showing lower spindle density than m0/m3, while slow-oscillation density also differed (χ^2^ = 16.20, p = 3.04×10^−4^, q = 3.69×10^−4^) and m6 showed stronger slow-oscillation morphology. Thus, SSL-defined N2 substates capture physiologically interpretable within-stage NREM structure, not merely stage duration or stage admixture.

Collectively, the microstate and meta-state analyses show how SSL extends, rather than replaces, the conventional stage scaffold. Canonical stages explain substantial structure and remain a strong baseline, but SSL embeddings preserve additional stage-anchored variation that is collapsed by five-stage summaries. We therefore interpret the recovered microstates and meta-states as operational summaries of representational structure, not as an ontologically fixed set of sleep states. Their value is instead supported by downstream prediction, robustness analyses, and physiological anchoring: within-stage heterogeneity provides measurable information beyond stage-only summaries, with N2-linked structure contributing most clearly to apnea- and BMI-related analyses and age-related signal distributed more broadly across stage-linked structure.

## Discussion

This study shows that self-supervised learning (SSL) foundation models trained on unlabeled sleep EEG preserve health-informative structure that is partially collapsed by conventional five-stage scoring. Prior sleep foundation-model studies have demonstrated strong predictive performance, but it has remained unclear whether such gains mainly repackage conventional sleep architecture or reflect finer-grained physiology beyond the hypnogram. By comparing SSL with architecture-matched from-scratch and five-stage-supervised models, we show that the five-stage interface remains a strong clinical scaffold rather than a strawman. At the same time, SSL retains additional information, most clearly for age and BMI and more modestly for AHI, suggesting that learned representations preserve stage-anchored physiological structure beyond the conventional stage labels.

These findings extend a long literature arguing that sleep physiology is more continuous and heterogeneous than the five-stage hypnogram implies. Earlier automated quantification and sleep/wake continuum approaches showed that sleep structure could be represented with finer gradations than visual stage labels^20,21^. More recent clustering and embedding studies similarly showed that data-driven sleep states map onto, but also subdivide, conventional stages and relate to traits such as age and apnea^9,25,44^. This study’s contribution is to test these ideas in a matched foundation-model framework, showing that SSL embeddings recover the canonical stage scaffold without labels while preserving additional stage-anchored structure beyond it.

Head-to-head comparisons show that SSL pretraining robustly outperforms from-scratch training, whereas advantages over the matched five-stage representation were narrower and outcome-specific. The clearest EEG-only advantages were for age and BMI, with smaller or nonuniform evidence for AHI and other outcomes. Expanded controls further showed that SSL was not merely rediscovering conventional sleep architecture or classical spectra. Spectral summaries were informative, especially for age, and the five-stage representation was competitive, but SSL retained incremental value beyond covariates, stage summaries, spectral summaries, and the matched five-stage representation. Thus, the interpretation is not that SSL signal is independent of known EEG physiology; rather, SSL organizes partly familiar spectral and NREM microphysiological information in a higher-resolution, task-relevant representational space.

To interpret this added information, we mapped SSL embeddings back to the canonical stage scaffold using stage-anchored cross-subject meta-states. These analyses do not support a fixed new sleep-state taxonomy; instead, they show that SSL preserves health-informative microstructure within familiar stages. N2 provides the clearest example, though not the only source of signal. Stage-conditioned meta-state analyses indicate that N2-linked structure preserves substantial apnea- and BMI-related information, whereas age-related signal is distributed more broadly across Wake/N1-, REM-, and N2-linked structure. The N2-linked meta-states also showed distinct and sometimes opposing phenotype associations, explaining how collapsing all N2 epochs into a single label can attenuate or cancel health-relevant signal. Finally, these N2 subdivisions were physiologically anchored: within AASM-scored N2, they differed in low-frequency/theta and sigma structure, and spindle/slow-oscillation analyses distinguished more spindle-rich N2 motifs from a spindle-sparse motif with stronger slow-oscillation morphology. Thus, SSL appears to preserve within-stage NREM microphysiology that conventional staging compresses, rather than replacing staging or localizing all added information exclusively to N2.

For digital medicine, these findings position EEG-only SSL representations as compact, reusable summaries of overnight physiology for screening, triage, and biomarker discovery. Their value is not in replacing the five-stage hypnogram, but in preserving that familiar scaffold while adding low-dimensional, stage-anchored summaries of within-stage physiology. Meta-state occupancy profiles could therefore be reported alongside conventional sleep summaries, while embedding manifolds may provide a coordinate system for visualizing how diagnostic or trait-related variables align with specific regions of sleep physiology.

The age result illustrates this translational logic. Although chronological age is directly observed, EEG-predicted age can be interpreted as a probe of age-sensitive neurophysiology and as the basis for brain-age-gap biomarkers. Future work should test whether Sleep2.0-derived age residuals predict cognitive decline, dementia risk, or other aging-related outcomes beyond chronological age and conventional sleep summaries. Similarly, EEG-derived apnea scores showed screening-oriented discrimination, calibration, and decision-analytic behavior, but these analyses are intended for participant-level decision support / risk stratification, not as replacements for event-based respiratory scoring, which remains the diagnostic standard.

Several limitations guide clinical translation. First, these retrospective analyses require prospective validation in real-world clinical and home-monitoring settings. Second, our EEG-only analyses used central PSG EEG rather than frontal consumer headband montages; thus, their home-monitoring relevance is translational rather than direct validation of consumer frontal EEG. Wearable EEG, wrist PPG, actigraphy, and HSAT/polygraphy are complementary sensing regimes, and respiratory-event detection from airflow, effort, oximetry, or PAP flow remains the clinical substrate for apnea diagnosis. Third, self-supervised models may encode biases from their training cohorts, requiring population-aware validation across age, sex, race/ethnicity, disease burden, acquisition setting, and sensor configuration.

Methodologically, the microstate/meta-state framework presented here is just one interpretable summary of embedding structure, not the only one possible. Direct pooled clustering of all epochs across subjects is a reasonable alternative, but it answers a different question: it estimates a single cohort-level partition, whereas our two-step approach first protects and compresses recording-specific microstructure and then aligns those local substates into a shared cross-subject vocabulary. The present analyses provide an initial physiological anchor for this vocabulary by showing that N2-linked meta-states differ in spectral profile, spindle expression, and slow-oscillation density and morphology within AASM-scored N2. Future work should extend event-level validation to K-complexes, spindle–slow-oscillation coupling, arousal and respiratory-event timing, richer EEG topographies, independent event detectors, and external cohorts.

Finally, our results should not be interpreted as requiring this specific HuBERT-style transformer. Smaller 2-layer and 6-layer models showed qualitatively similar patterns, but the matched SSL versus five-stage comparison indicates that the pretraining objective –stage supervision versus self-supervision– matters. Future studies should compare HuBERT-style masked prediction with contrastive and generative objectives, and use attribution or probing analyses to identify the signal features supporting each outcome.

Ultimately, foundation model-based sleep analytics will require clear intended-use definitions—such as screening enrichment, decision support, or biomarker discovery—rather than claims of full diagnostic replacement. By reducing reliance on expert labels while remaining aligned with clinically familiar concepts, self-supervised representations offer a scalable route toward continuous, data-driven health assessment from passive sleep recordings.

## Methods

### Data sources, partitions, and outcomes

We assembled a pretraining corpus of 11,261 overnight polysomnography (PSG) records from 7,201 participants by combining seven large, publicly available datasets hosted by the National Sleep Research Resource (NSRR): MESA, MrOS, SHHS, WSC, SOF, CFS, and NCHSDB.^49–56^ All recordings from 779 randomly selected participants (~10% of the pretraining cohort) were held out as an internal validation partition for model selection.

To evaluate generalization, we additionally held out three external benchmark datasets—Dreem Open^57^, Sleep-EDF^58,59^, and HomePAP^60^—that were never used during pretraining. For downstream fine-tuning and clinical/digital-medicine evaluation, we focused on APPLES (Visit 3)^61,62^, which couples PSG with rich baseline physiology and standardized cognitive and mood assessments. Outcomes analyzed in APPLES comprised diagnostic sleep metrics (five-stage sleep staging, apnea–hypopnea index [AHI], Epworth Sleepiness Scale [ESS]^63^), demographic variables (age, sex), and functional measures spanning mood and cognition (BDI^64^, HAM-D^65^, POMS^66^, WASI-IQ^67^, Pathfinder^68^, MMSE^69^, Buschke^70^, SWMT^71^, and PVT^72^). All experiments used strict participant-level splits (all segments and all nights for a given participant assigned to a single fold) to prevent information leakage across training and evaluation.

### Signal preprocessing and model inputs

PSG signals were harmonized across datasets using a common preprocessing pipeline. We extracted a central EEG derivation (C3 or C4) as the core modality and additional PSG channels (EOG, chin EMG, ECG, and thoracic respiratory effort) for multimodal experiments. Signals were resampled to 100 Hz, robustly normalized by median-centering and IQR-scaling after excluding values beyond 20 IQR^12^, and segmented into 30 s epochs consistent with AASM conventions. For transformer inputs, we concatenated 101 consecutive epochs (50.5 min) into fixed-length sequences. During pretraining, sequences were sampled with overlap (hop size 25 epochs); during fine-tuning and evaluation, sequences were sampled without overlap.

### Self-supervised pretraining and matched baselines

We trained transformer foundation models on unlabeled sleep recordings using a HuBERT-style masked pseudo-label prediction objective. In stage 1, each 101-epoch input sequence was converted to epoch-level time-frequency power features, and mini-batch k-means with k = 100 was used to assign discrete pseudo-labels. The model was trained from raw input signals to predict pseudo-labels for masked portions of the sequence, learning contextual representations without stage labels. In stage 2, pseudo-labels were recomputed from first-stage transformer embeddings at the model’s native temporal resolution, yielding 946 labels per 50.5-min sequence, and clustered with k = 500 before retraining from the first-stage checkpoint with an updated output layer.

All downstream comparisons used two architecture-matched baselines: (i) a supervised pretraining baseline optimized to predict conventional five-stage sleep labels, and (ii) a no-pretraining control initialized randomly and trained directly on each downstream task. For downstream fine-tuning, the pretraining output layer was replaced with a task-specific regression or classification head, and the full network was fine-tuned end-to-end using participant-level cross-validation.

### Fine-tuning experiments and statistical testing

To probe health information encoded in the representations, we fine-tuned each pretrained (or scratch) model on APPLES for each outcome. Regression performance was evaluated by Pearson correlation (r) and classification performance by accuracy. We used participant-level 10-fold cross-validation: within each fold, participants were split into training (80%), validation (10%), and test (10%) subsets, and the best validation checkpoint was evaluated on the held-out test subset. Pairwise representation comparisons (SSL vs from-scratch; SSL vs five-stage pretraining) used paired, fold-wise tests (Wilcoxon signed-rank). Nominal p-values were adjusted across the 15 APPLES outcomes using Benjamini–Hochberg false discovery rate (FDR) control and are reported as q-values (q < 0.05).

### Digital medicine evaluation: screening, calibration, and confound controls

To evaluate participant-level screening rather than epoch-level prediction, we generated multiple segment-level predictions per participant and summarized each participant by the mean model output across segments. For binary screening tasks, continuous outcomes were thresholded using established clinical cutoffs: AHI ≥ 15 and AHI ≥ 30, BMI ≥ 30, age ≥ 60 years, and sex; additional functional thresholds used in exploratory analyses are listed in Supplementary Table 1. Screening discrimination was quantified using ROC curves and AUROC.

For severe-apnea screening (AHI ≥ 30), we additionally calibrated model scores to predicted probabilities using out-of-fold Platt scaling and evaluated calibration with reliability curves and Brier scores; clinical utility was assessed via decision curve analysis (DCA) against treat-all and treat-none baselines.

To test whether SSL-derived signals provided incremental information beyond simpler explanatory baselines, we conducted nested participant-level control analyses using four non-overlapping feature sources: clinical/demographic covariates, conventional stage-derived sleep-report summaries, conventional EEG spectral summaries, and target-specific scores from either the SSL model or the matched five-stage-pretrained model. Covariates included age, sex, BMI, and AHI with outcome-specific exclusions to avoid circularity. Stage summaries included standard sleep-architecture features such as stage proportions, sleep efficiency, wake after sleep onset, bout/transition statistics, and related hypnogram-derived measures. Spectral summaries included conventional whole-night and NREM EEG features, including absolute and relative band power, slowing ratios, spectral centroid, median/edge frequency, and aperiodic 1/f parameters. We evaluated covariate-only, stage-only, spectra-only, model-score-only, covariate + stage, covariate + stage + spectra, and fully adjusted models that added either the SSL score or the matched five-stage-model score to the same covariate + stage + spectra baseline. SSL and five-stage scores were not entered together, allowing a direct comparison of each learned representation against identical non-transformer baselines. Models were evaluated with participant-level 5-fold cross-validation; fold-wise AUROC differences were tested using exact paired sign-flip tests with FDR correction within each comparison family. For continuous outcomes, we additionally assessed incremental residual signal by correlating SSL and five-stage scores with out-of-fold residuals after regressing out covariates, stage summaries, and spectral summaries (Spearman ρ).

### Representation structure analyses and meta-state framework

To interpret what the models learn beyond traditional staging, we analyzed epoch-level embeddings from the final transformer layer. Because transformer embeddings are contextual over the 101-epoch input sequence, each embedding was indexed to the AASM label of the corresponding focal 30-s epoch. These labels were used as anchors for interpretation, not as evidence that the embedding reflected only that isolated epoch. Stage-composition summaries were computed by aggregating focal-epoch AASM labels over embeddings assigned to each microstate or meta-state. Embedding geometry was visualized with t-SNE/UMAP and related to expert stage labels and participant phenotypes. To quantify within-night structure, we performed per-recording k-means clustering on embeddings and selected the number of clusters (“microstates”) using Bayesian Information Criterion (BIC) sweeps. We then introduced a meta-state framework to summarize microstructure across participants: subject-specific microstates were pooled and clustered into a consistent set of M meta-states, and each participant was represented by meta-state occupancy and diversity features. We used this two-step microstate-to-meta-state procedure because the first step estimates recording-specific within-night structure, including per-recording state granularity, whereas the second step aligns those local substates into a shared cross-subject vocabulary. Direct pooled clustering would instead impose a single global partition and fixed granularity on the cohort, and would be more strongly driven by the most prevalent epochs/stages and longest recordings.

To avoid circular selection of M, we implemented nested cross-validation: an inner loop selected M over a sweep (M = 3–15) using a stability-constrained performance-plateau criterion, and an outer loop evaluated downstream prediction using the chosen meta-state features. We tested whether meta-state features improved prediction beyond stage-only summaries by training linear models on (i) stage-only features and (ii) stage summaries augmented with meta-state occupancy/diversity, reporting mean out-of-fold AUROC (binary) or R^2^ (continuous). To localize incremental signal to canonical stages, we computed stage-conditional meta-state occupancies (e.g., within-N2 or within-Wake occupancy independent of total time in that stage) and conducted dominant-stage ablations in which meta-states dominated by N2 and/or Wake were selectively excluded.

To assess robustness to modeling choices, we repeated the clustering-based analyses under controlled perturbations. For the per-record microstate step, each recording’s selected k was held fixed and k-means was rerun across 10 random initializations to quantify assignment recoverability (ARI/AMI) and stage-anchored Rosetta-count stability. For the cross-subject meta-state step, the APPLES M = 7 solution was rerun across 10 seeds; local sensitivity was additionally assessed over M = 5–9 and PCA dimensionality of 10, 20, and 50. A fixed-k sensitivity analysis near the empirical SSL median was used to verify that qualitative Rosetta/meta-state conclusions were not dependent on BIC-selected k. Preprocessing was held fixed across matched analyses to preserve representation-level comparability.

Analyses were implemented in Python (NumPy, pandas, scikit-learn, PyTorch).

## Supplementary Material

Supplementary Files

This is a list of supplementary files associated with this preprint. Click to download.

- SupplementaryTable1.png
- SupplementaryTable2.png
- SupplementaryTable3.pdf
- SupplementaryFiguresandTables.pdf

## Data Availability

All datasets used in this study are publicly available. Model pretraining was performed using large-scale publicly available polysomnographic datasets accessed through the National Sleep Research Resource (SleepData.org), including MESA, MrOS, SHHS, WSC, SOF, CFS, and NCHSDB, comprising a total of 11,261 overnight recordings (Supplementary Table 3). Model evaluation was conducted on held-out datasets not used for pretraining, including APPLES and HomePAP (SleepData.org), as well as the Dreem Open dataset and Sleep-EDF, which are available via PhysioNet. Access to these datasets is subject to dataset-specific data use agreements and approval procedures. Because redistribution of raw polysomnographic recordings is governed by these agreements, we do not host raw data; however, all preprocessing and dataset construction scripts are provided to enable independent reconstruction of the analytic cohorts from the original sources.

## Code Availability

All code used for data preprocessing, model training, evaluation, and figure generation, along with trained model weights, is publicly available via the Open Science Framework (OSF) at https://osf.io/j2mkh/. The repository includes documentation sufficient to reproduce the analyses reported in this study.
